# Synergistic Activity of Deguelin and Fludarabine in Cells from Chronic Lymphocytic Leukemia Patients and in the New Zealand Black Murine Model

**DOI:** 10.1371/journal.pone.0154159

**Published:** 2016-04-21

**Authors:** Nerea Rebolleda, Ignacio Losada-Fernandez, Gema Perez-Chacon, Raquel Castejon, Silvia Rosado, Marta Morado, Maria Teresa Vallejo-Cremades, Andrea Martinez, Juan A. Vargas-Nuñez, Paloma Perez-Aciego

**Affiliations:** 1 Fundacion LAIR, Madrid, Spain; 2 Instituto de Investigaciones Biomedicas “Alberto Sols”, CSIC-UAM, Madrid, Spain; 3 Servicio de Medicina Interna, Hospital Universitario Puerta de Hierro Majadahonda, IDIPHIM, Universidad Autonoma de Madrid, Madrid, Spain; 4 Servicio de Hematologia y Hemoterapia, Hospital Universitario La Paz, Madrid, Spain; 5 Laboratorio de Imagen, Plataforma Apoyo a la Investigación, IdiPaz, Hospital Universitario La Paz, Madrid, Spain; University of Manitoba, CANADA

## Abstract

B-cell chronic lymphocytic leukemia (CLL) remains an incurable disease, and despite the improvement achieved by therapeutic regimes developed over the last years still a subset of patients face a rather poor prognosis and will eventually relapse and become refractory to therapy. The natural rotenoid deguelin has been shown to induce apoptosis in several cancer cells and cell lines, including primary human CLL cells, and to act as a chemopreventive agent in animal models of induced carcinogenesis. In this work, we show that deguelin induces apoptosis *in vitro* in primary human CLL cells and in CLL-like cells from the New Zealand Black (NZB) mouse strain. In both of them, deguelin dowregulates AKT, NFκB and several downstream antiapoptotic proteins (XIAP, cIAP, BCL2, BCL-X_L_ and survivin), activating the mitochondrial pathway of apoptosis. Moreover, deguelin inhibits stromal cell-mediated c-Myc upregulation and resistance to fludarabine, increasing fludarabine induced DNA damage. We further show that deguelin has activity *in vivo* against NZB CLL-like cells in an experimental model of CLL in young NZB mice transplanted with spleen cells from aged NZB mice with lymphoproliferation. Moreover, the combination of deguelin and fludarabine in this model prolonged the survival of transplanted mice at doses of both compounds that were ineffective when administered individually. These results suggest deguelin could have potential for the treatment of human CLL.

## Introduction

B cell chronic lymphocytic leukemia is characterized by a progressive accumulation of monoclonal mature CD5^+^ B cells within lymphoid organs and in the peripheral blood. In the last decade, development of chemoimmunotherapy combining purine nucleoside analogs, like fludarabine [1], with monoclonal antibodies targeting CD20 [2] has improved significantly the outcome for patients with CLL, and currently the gold standard of first-line treatment is the combination of cyclophosphamide, fludarabine and Rituximab (FCR) [3]. However, this treatment is not feasible for some patients due to advanced age and/or comorbid conditions, and thus better tolerated alternatives are needed for such cases. Also, a subset of treated patients will eventually relapse and develop resistance to the cytotoxic therapy [4]. The PI3K/AKT/NFκB pathway plays a key role in maintaining survival of CLL cells [5–8], and is also involved in acquisition of microenvironment-mediated resistance to purine analogs like fludarabine [9–11]. A number of compounds that target protein kinases involved in the integration of signals coming from the microenvironment have been tested over the last years and shown activity *in vitro* against CLL cells [12]. Two of them, the PI3Kδ inhibitor Idelalisib and the BTK inhibitor Ibrutinib, have demonstrated good efficacy with tolerable doses, and have been recently approved by the FDA for their use in relapsed or refractory CLL [13,14], providing evidence that downregulation of protein kinases important for tumor cell survival can be a useful strategy for the treatment of CLL.

Deguelin, a natural rotenoid isolated from Leguminosae plants like *Mundulea sericea* [15], has been shown to be effective in reducing the growth of several human tumor cell lines, both *in vitro* [16,17] and in tumor xenograft models such as breast or lung carcinomas [18,19]. Deguelin is a potent inhibitor of HSP90 [20,21] and mitochondrial bioenergetics [22], and decreases the activity of the AKT/NFκB pathway [23] leading to a reduced expression and function of several NFκB-regulated genes involved in cell survival and proliferation, such as BCL2, BCL-X_L_, XIAP, cIAP, survivin, CCND1 (cyclin D1), CCNE (cyclin E), or CDK4 [16,19,24,25]. Deguelin has been shown to promote apoptosis of CLL cells, promoting downregulation of IκBα [26]. In this work, we extend on previous findings regarding deguelin action in CLL cells, and evaluate deguelin as a potential therapeutic compound in CLL in an *in vivo* model in the NZB mouse strain. By one year of age, virtually all NZB mice display a clonal hyperplasia of B220^low^ CD5^low^ IgM^+^ hyperdiploid cells that are first detected in the peritoneal cavity and progressively colonize the spleen, occasionally progressing to a lymphoid neoplasia that infiltrates other lymphoid and non-lymphoid tissues like bone marrow, lymph nodes, liver or lungs [27]. This leukemic cells have many similarities to the malignant cell in human CLL [28,29]. Earlier reports described that spleen cells from aged NZB mice with a hyperdiploid leukemic cell population can be serially passaged into young NZB or NZB/DBA2 F1 recipients, reproducing the pattern of growth and spreading of the spontaneous-arising leukemic cells and becoming the dominant B cell population [30,31]. By transplanting leukemic cells from aged NZB mice with splenomegaly into young NZB recipients, development of CLL-like disease was anticipated and synchronized in the transplanted animals, presenting with virtually complete penetrance, thus making it a model suitable for studying the efficacy of experimental treatments for CLL. We show that deguelin induces apoptosis in cells from CLL patients by inhibiting the AKT/NFκB pathway. Also, deguelin shows activity *in vivo* against the CLL-like cells in the NZB mouse strain. In combination with fludarabine, deguelin remarkably potentiates fludarabine-induced dsDNA strand breaks showing a mild synergistic effect *in vitro* on human CLL cells and NZB CLL-like cells, and prolongs survival in an experimental model of CLL established by transfer of splenocytes from old NZB mice with leukemic hyperdiploid CD5^+^ B cells into young NZB recipients.

## Methods

### Ethics Statement

Study design, collection of blood samples from all participants, and analysis of clinical data from patients were approved by the Ethical Review Committee (Hospital Puerta de Hierro, Majadahonda, Permit N° 313). Written informed consent was obtained from healthy donors and 7 CLL patients. An oral informed consent to participate in the research was obtained from another 28 CLL patients after receiving a verbal summary of the project. All procedures regarding the use of human biological samples, including the use of an oral consent form, were approved by our institutional Ethical Review Committee.

All animal procedures were approved by the Universidad Autonoma de Madrid (UAM) institutional Animal Care and Use Committee (Permit N° ES280800000012), and carried out in compliance with both national and European legislation (RD 53/2013 and EU Directive 2010/63/EU) for the protection of animals used for research experimentation and other scientific purposes, and was reviewed and approved by UAM Institutional Animal Care and Use Committee.

### CLL patient and control samples

Peripheral blood was collected from 35 CLL patients and 10 healthy control donors in 10 ml heparinized tubes. Peripheral blood mononuclear cells (PBMCs) were isolated by gradient centrifugation (Lymphocytes Isolation Solution, Comercial Rafer). The main clinical and analytical parameters of the patients are shown in S1 Table. CD38 and ZAP-70 expression were quantified by flow cytometry as previously described [32]. All patients had been untreated at least for 3 months before blood collection.

### Mutation status of the variable region of the immunoglobulin heavy chain gene

Total RNA was extracted from peripheral blood mononuclear cells of CLL patients using the High Pure RNA Isolation Kit (Roche Diagnostics). 1–5 μg RNA were reverse-transcribed using AMV reverse transcriptase (Roche Diagnostics). Reactions were carried out in 10 μl volume using 5 mM MgCl_2_, 3.2 μg random primers, 1 mM dNTPs and 20 U of AMV. IgVH products were amplified by PCR in a 50 μl reaction with 1.5 mM MgSO_4_, specific primers [33], 0.2 mM dNTPs, and 1.5 U of Pwo DNA polymerase (Roche Diagnostics), following a previously described amplification program [34]. PCR products were isolated and sequenced directly using an automated sequencing system (ABI 31030XL from Applied Biosystems) as previously described [35]. Sequences obtained were compared with reference sequences of immunoglobulin germline genes available at the international ImMunoGenetics information system® using the IMGT/V-QUEST tool (http://imgt.cines.fr/IMGT_vquest/share/texts/) [36]. We defined as “unmutated IgVH”(U) those sequences with ≥98% similarity with the reference sequences, and “mutated IgVH” (M) those with <98% similarity.

### Mice

Female young (4–6 week) and old (9 months) NZB mice (NZB/Ola Hsd) were purchased from Harlan Laboratories and housed under specific pathogen free conditions at the UAM university animal facility (Animalario, Facultad de Medicina, UAM). Four mice were randomly allocated per cage, and maintained in a 12 h light/12 h dark cycle with controlled temperature and humidity with provision of nesting material and access to food and water *ad libitum*. Mice were supervised daily by the animal facility staff, and monitored by us 2 days/week, or daily when interventions were performed. Animals were euthanized in a CO_2_ chamber, following established criteria to determine humane end points. End points were used for animals included in the survival study, and consisted in the appearance of signs of morbidity, such as ruffled fur, hunched back, rapid breathing or lethargy. Weights were weekly recorded, and a weight loss of >20% sustained for a week was also considered an end point. In the vast majority of cases, mice performed well until they simultaneously showed several signs of distress that fulfilled the end point criteria. Sporadic cases of dehydration without any other sign of distress were treated with subcutaneous injections of 0.2 ml saline, and daily monitoring of skin appearance and mouse behavior. Superficial wounds were treated with iodine solution and monitored for wound healing.

### Experimental model of CLL in transplanted NZB mice

An experimental model of CLL was established in the mouse NZB strain by transfer of cells from enlarged spleens of aged (>1 year) NZB females into young (4–6 week) female NZB recipients. Spleen cells from three aged diseased female NZB mice were pooled, and 20 x 10^6^ cells per mice were transplanted intraperitoneally (i.p.) into 40 young NZB recipients. Two months after transplants, growth of transplanted leukemic cells was assessed by detection of hyperdiploid B220^low^ CD5^low^ IgM^+^ cells in the peripheral blood by flow cytometry. The enlarged spleen from one of the transplanted mice was subsequently transplanted i.p. into five young NZB mice used to study the effect of deguelin on CLL-like cells *in vivo*. Drug treatments in mice consisted on 4 mg/kg deguelin dissolved in corn oil given intragastrically (i.g.) and/or 35 mg/kg fludarabine in saline phosphate (Hospira Pharmaceuticals) given intraperitoneally (i.p.).

### Mouse tissue samples

Peripheral blood from mice was obtained from the submandibular vein in 0.5 ml EDTA tubes (Greiner bio-one). Immediately after euthanizing the mice, blood was collected from the hearts. Spleen, liver, gut, femurs, lymph nodes, kidneys and lungs were removed for cell isolation and/or tissue fixation. Spleen cells were obtained by mechanically disrupting the tissue in fresh prewarmed culture medium with 20 U/ml DNAse I (Roche Diagnostics), and mononuclear cells were isolated by gradient centrifugation using Lympholyte-M (Cedarlane Laboratories). Tissue samples for immunohistochemistry were prepared by fixing in neutral buffered formalin for 24–48 h, dehydration and inclusion in paraffin.

### Immunohistochemistry

Paraffin-embedded tissue sections were deparaffinized, rehydrated and stained with hematoxylin and eosin. For immunostaining, 5 μm thick sections were unmasked, endogenous peroxidase was blocked with 0.1% H_2_O_2_ diluted in methanol for 10 min and unspecific antibody binding was blocked with 1% BSA, 1% triton X-100, 2% horse serum in Tris buffer for 1–2 h. Tissues were stained overnight at 4°C with primary antibodies in blocking solution and visualized with Dako Real™ detection system AEC K5003 (Dako) following manufacturer's instructions. The following primary antibodies were used: anti-phospho-AKT (Ser^473^), anti-p65 NFκB subunit (E498) and anti-IκBα (L35A5) from Cell Signaling Technologies; anti-survivin and anti-cyclin D1 from Sigma. Nuclei were counterstained with hematoxylin, and tissue sections were visualized in an Olympus BX14 microscope, with 100x, 200x and 400x total magnification, and analyzed with the Image proPlus software (Media Cybernetics).

### Immunoblotting

Western blots were done as previously described [37], with the primary antibodies: anti-AKT (11E7), anti-phospho-AKT(Ser^473^), anti-p65 NFκB subunit (E498), anti-phospho-p65 (Ser^536^), anti-IκBα (L35A5), anti-caspase 3,anti-caspase 9, anti-c-Myc and anti-phospho-GSK3αβ (Ser^21,9^) from Cell Signaling Technology; anti-BCL2, anti-BAX (6a7), anti-survivin, anti-cyclin D1 and anti-β-actin (AC-15) from Sigma; anti-BCL-X_L_ and anti-PARP (C2-10) and anti-p53 from BD Biosciences; anti-phospho-histone H2A.X (Ser^139^) and anti-cIAP (315301) from R&D Systems and anti-GSK3β from Santa Cruz Biotechnologies.

### Cell culture and treatments

Freshly isolated human PBMCs or murine spleen cells were cultured at 10^6^/ml in RPMI 1640 medium with 10% heat-inactivated fetal bovine serum, 2 mM L-glutamine, 10 U/ml penicillin and 100 μg/ml streptomycin (all from Life Technologies) at 37°C in an humidified 5% CO_2_ atmosphere. Cells were cultured either alone or over a monolayer of confluent murine *Ltk*^*-*^ fibroblasts (ATCC) or a derivative of the 3T3 cell line that constitutively expresses the human CD40LG gene [38] as feeders. The day before coculturing with mononuclear cells, monolayers were treated with 50 μg/ml mitomycin C (Sigma), washed 3 times in RPMI 1640 medium, and seeded in 24-well plates. Deguelin (Tocris Bioscience) was dissolved in DMSO at 10 mM as a stock solution and working solutions were prepared from the stock in RPMI 1640 medium. Deguelin treated cells were preincubated 1h at 37°C with 0–100 μM deguelin before culturing alone or over feeder cells. Control cells received 0.1% DMSO in complete medium (the maximum final DMSO concentration in deguelin treated cells). Fludarabine (F-ara-A, Sigma) was dissolved in DMSO at 10 mM as a stock solution and working solutions were prepared from the stock in RPMI 1640 medium. 0–50 μM fludarabine was added to the cells after plating them alone or with feeder cells.

Doses causing apoptosis in 50% of cells (ED50) in deguelin or fludarabine treated cells were calculated from dose-response curves by non-linear regression fit using GraphPad 5.0 (GraphPad Prism Software). The interaction of deguelin plus fludarabine was studied by adding both drugs at constant molar ratios of 1:2 over the range of concentrations indicated above and cytotoxicity of the combination was calculated by the Chou–Talalay method with the CalcuSyn software (Biosoft) [39]. Combination index (CI), computed from dose–effect curves of drugs alone and in combination, represents a measure of the effect of drug interaction (additive effect: = 1, synergism: ≤ 0.9and antagonism: ≥ 1). As CI depends on the ‘fractional effect level’ at which it is calculated we considered three levels of cytotoxicity, ED50, ED75 and ED90 (concentration lethal to 50%, 75% and 90% of CLL cells). Dose reduction index (DRI), calculated from cytotoxicity profiles of drugs alone and in combination, indicates how many times fludarabine doses can be reduced, when used in combination with a coordinated dose of deguelin, to reach a given cytotoxicity.

### Flow cytometry

Cell viability in deguelin and fludarabine treated cells was assessed from cultured cells by staining with Annexin V-FITC (Roche Diagnostics)/Propidium Iodide (PI)(Sigma). For caspase inhibition, 50 μM z-VAD-fmk (benzyloxy-carbonyl-Val-Ala-Asp-fluoro-methylketone, Tocris Biosciences) was added to cultures 1 h prior to the addition of drugs. Cytotoxicity in subpopulations of PBMCs was assessed by surface staining with fluorochrome-conjugated monoclonal antibodies to CD3 (BD) and CD19 (BD), and then fixation and permeabilization with the Fix&Perm® kit (Life Technologies) plus intracellular staining with Alexa-Fluor 488® phalloidin (Life Technologies).

For intracellular staining we used purified rabbit antibodies directed to phosho-AKT (Ser^473^) (98H9L8, Life Technologies); c-Myc, phospho-p65 (Ser^536^) and phospho-H2AX (Ser^139^, 20E3) from Cell Signaling Technology, and a Goat anti-rabbit IgG conjugated with Alexa-Fluor 488® (Life Technologies). Cells were permeabilized following a protocol available from Cell Signaling Technology (Combined Staining of Intracellular Proteins and Cell Surface Markers in Blood)

Changes in the mitochondrial transmembrane potential (ΔΨm) were measured in drug-treated cells by staining with 7.6 μM JC-1, (5’,6,6’-tetrachloro-1,1’,3,3’-tetraethylbenzimidazolylcarbocyanine iodide, Sigma) for 15 min following manufacturers’ instructions, followed by detection of JC-1 red fluorescence emission and quantification of the fraction of events in the low red fluorescence peak. As a depolarization control, cells were pre-incubated in the presence of 50 μM CCCP (carbonyl cyanide 3-chlorophenylhydrazone, Sigma) for 1 h before JC-1 staining.

Mouse leukemic cells were also detected in mononuclear cell samples from blood or spleen by staining for IgM, B220 and CD5 with fluorescent-conjugated antibodies (all from BD Biosciences). Hyperdiploid NZB leukemic cells were detected as previously described by DNA staining with Propidium iodide and cell cycle analysis [40] with the Modfit LT software (Verify Software House). Flow cytometry analyses were performed on a FACSort flow cytometer (BD Biosciences) using the CellQuest® software (BD Biosciences).

### Statistical analysis

Statistical analysis was carried out using GraphPad 5.0 (GraphPad Prism Software, Inc). Unpaired and paired tests were used to assess differences between groups. Non-linear dose- response curves were fit using a four-parameter logistic regression model. Survival fractions were calculated by the product limit (Kaplan-Meier) method. P values less than 0.05 were considered statistically significant. Statistical power was calculated with the G-power3 software [41].

## Results

### Deguelin activates the mitochondrial pathway of apoptosis in primary CLL cells

**Fig 1:**
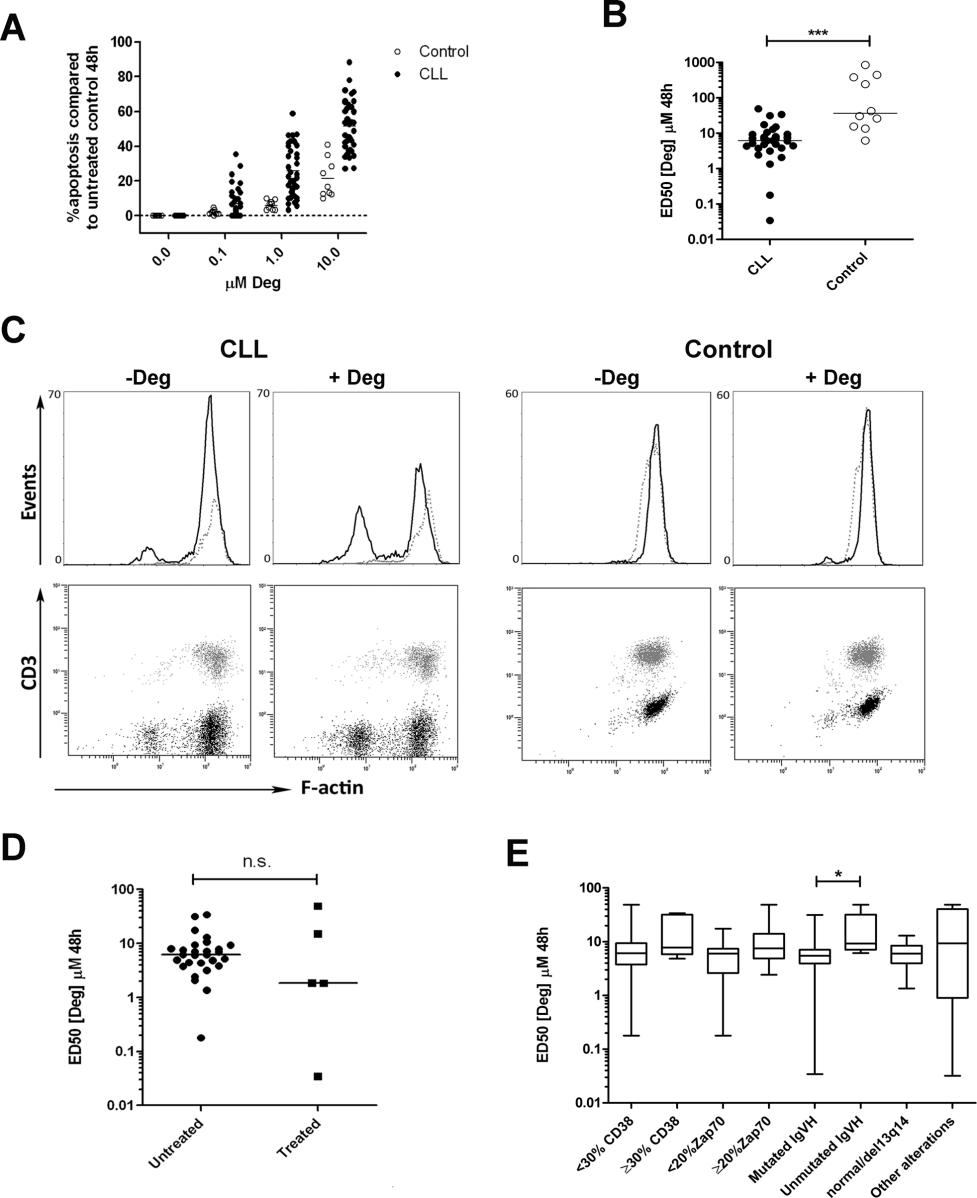
Deguelin induces cytotoxicity dependent on dose in primary CLL cells. PBMCs from CLL patients (n = 35) and healthy donors (n = 10) were incubated with or without deguelin for 48 h. Apoptosis was evaluated by flow cytometry after double staining with Annexin V-FITC and PI and it is expressed as a percentage relative to a time-matched untreated control containing diluent (0.1% DMSO). (A) Cells from patients and controls were exposed to increasing concentrations of deguelin (Deg). (B) A dose-response curve was constructed for each sample by non-linear regression fit, and the dose cytotoxic to 50% of the cells (ED50) was calculated. Medians are indicated by horizontal lines. *** P < 0.0005 (Mann-Whitney U test). (C) PBMCs from 5 CLL patients and 5 healthy controls were exposed to 10 μM deguelin (Deg) or diluent (-Deg) for 24 hours and then stained for surface CD3 and CD19 markers for T and B lymphocyte identification. After cell permeabilization, filamentous actin (F-actin) was stained with a fluorescence-conjugated phalloidin and analyzed by flow cytometry (representative samples are shown). Apoptosis was quantified as the loss of F-actin fluorescence in T (dotted line, grey dots) or B lymphocyte subpopulations (solid line, black dots). (D) ED50 values from patients were grouped into previously treated or untreated patients. (E) The same as in (D), in this case stratified by the prognostic markers: percentage of CD38 expression, ZAP70 expression, IgVH mutation status and the presence of cytogenetic alterations different from 13q14 deletions. Horizontal lines represent the median. * P < 0.05 (Mann-Whitney U test).

PBMCs from 35 CLL patients (>90% CD19^+^, CD5^+^ B cells) and 10 healthy donors were treated with increasing concentrations of deguelin (0–10 μM), and cell viability was evaluated at 48 h by Annexin V/PI staining and flow cytometry. Deguelin induced apoptosis in a dose-dependent manner (Fig 1A). For each sample, dose-response curves were plotted and the ED50 values were calculated. PBMCs from CLL patients had a significantly higher sensitivity compared to healthy donors (median ED50 values: 6.2 μM and 36.4 μM respectively, Fig 1B). Also, within 5 CLL samples treated with 10 μM deguelin for 24 h, CLL cells showed a higher sensitivity compared to normal T cells from the same patient (median % apoptotic cells (25–75 percentile): 23% (17.5–30) vs 0% (0–11), and compared to B (3% (0–5)) and T (0% (0–9)) cells from healthy controls (Fig 1C).

We then compared ED50 values for deguelin in samples from CLL patients stratified for treatment or markers associated with poor prognosis (Fig 1D and 1E and S1 Table). No significant differences were seen between treated or untreated patients or according to the presence of markers of worse prognosis (CD38, ZAP-70, cytogenetic alterations, IgVH mutational status), with the exception of IgVH mutational status, with unmutated IgVH CLL samples showing a higher resistance to apoptosis induced by deguelin (median ED50: 9 μM *vs* 5 μM, respectively). However, our CLL samples were limited in number, and biased with a majority having good prognostic markers, so the statistical power of these comparisons was low (0.26; 0.54; 0.64; and 0.35 respectively). Larger and more compensated groups will be needed to obtain more reliable comparisons.

**Fig 2:**
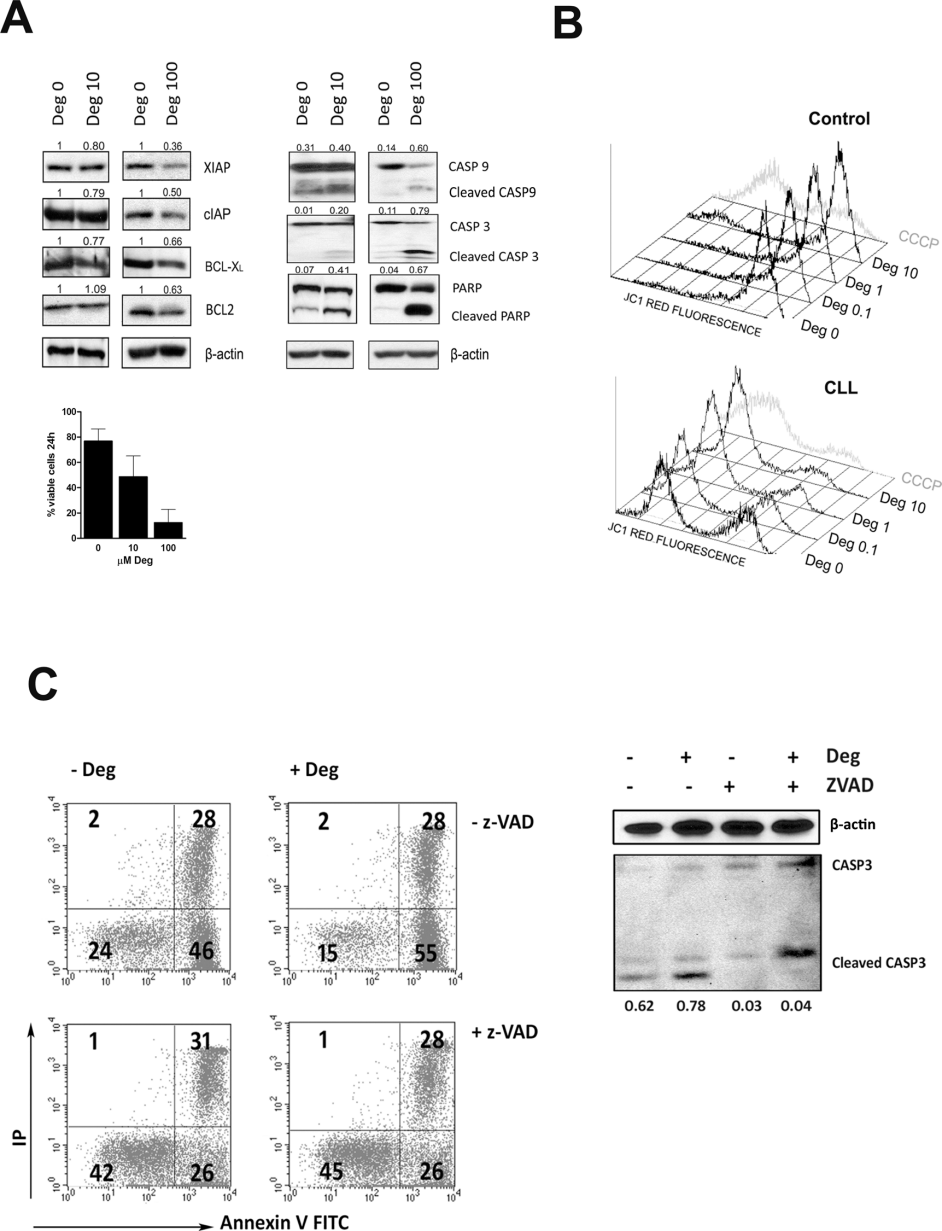
Deguelin reduces AKT signaling and induces apoptosis via the mitochondrial route in primary CLL cells. (A) PBMCs from 3 CLL patients were treated with 0, 10, or 100 μM deguelin and viability was evaluated by flow cytometry after Annexin V/IP staining at 24 h (bottom-right bar graphic). Cell lysates were then separated by SDS-PAGE, transferred onto PVDF membranes and analyzed by western blot. Immunoreactive bands for several deguelin target molecules and β-actin from a respresentative cell lysate were visualized with the ECL kit. A representative CLL sample is shown. Numbers indicate the signal intensity of each band from deguelin treated samples (Deg 10, Deg 100) compared to the untreated ones (Deg 0). For caspases (CASP3, CASP9) and PARP, the numbers indicate the proportion of cleaved fragments relative to the total protein (intact plus cleaved). (B) PBMCs from 3 CLL patients and 4 healthy controls were cultured for 24 h in the presence of up to 10 μM deguelin (Deg). The mitochondrial membrane potential was analyzed by flow cytometry after JC-1 staining for 15 min. As depolarization control cells were treated with 50 μM CCCP. Histograms show red fluorescence profiles in samples from a representative control and a CLL patient. (C) Cells from a representative CLL patient were treated with 50μM z-VAD-fmk in the presence or absence of 10 μM deguelin. After 16 h, the percentage of apoptotic cells was determined by Annexin V-FITC/PI staining and flow cytometry (left panels). Caspase 3 activation was assessed in protein lysates of treated cells by western blotting (right panel). The numbers indicate the proportion of cleaved fragments relative to the total protein (intact plus cleaved).

To search for specific apoptotic pathways induced by deguelin, PBMCs from 3 CLL patients were treated *in vitro* with 10 μM and 100 μM deguelin for 24 h. Deguelin dose-dependently downregulated the expression of several antiapoptotic proteins important in CLL cell survival (BCL2, BCL-X_L_, cIAP, XIAP, Fig 2A). Next, the effect on mitochondrial depolarization was examined in deguelin treated PBMCs from 3 CLL patients and 4 controls with the mitochondrial membrane potential sensitive dye JC-1. In line with the higher sensitivity to deguelin of CLL versus healthy B cells, 10 μM deguelin lead to mitochondrial membrane depolarization in a significantly higher percentage of PBMCs from CLL patients compared to healthy lymphocytes (54 ± 26% *vs* 24 ± 15% respectively, Fig 2B). Western blots in 10 μM and 100 μM treated cells show that mitochondrial membrane depolarization was accompanied by the cleavage of the initiator caspase 9, the final effector caspase 3 and its substrate PARP (Fig 2A). The mitochondrial pathway of apoptosis seemed to be the dominant mechanism of deguelin-induced cell death in our assays, as both caspase 3 activation and apoptosis were prevented by treatment with the pan-caspase inhibitor z-VAD-fmk (Fig 2C).

### Deguelin overcomes microenvironment derived pro-survival signals on CLL cells and acts synergistically with fludarabine

**Fig 3:**
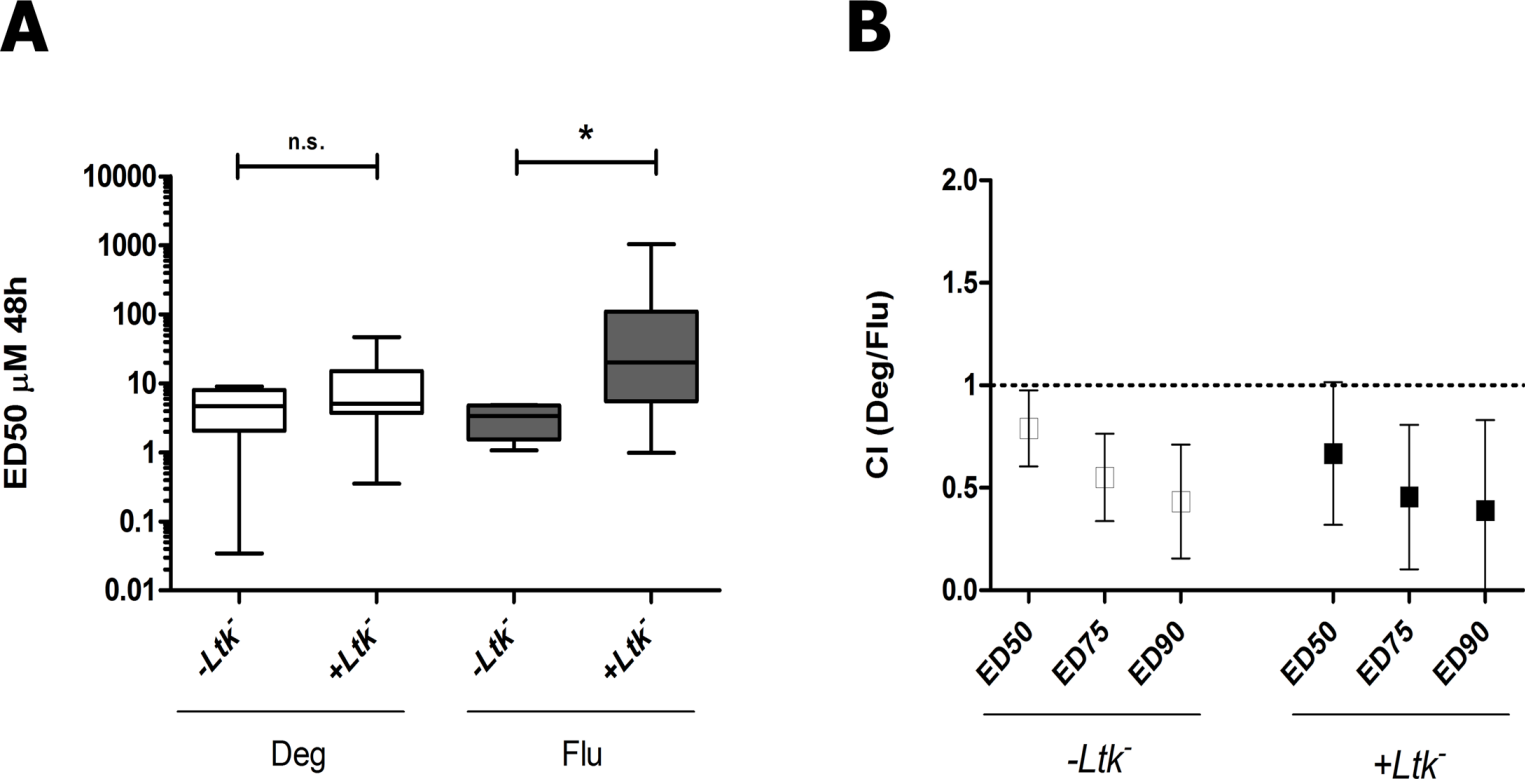
Deguelin overcomes microenvironment mediated pro-survival effects on primary CLL cells. Primary CLL cells (n = 8) were preincubated for 1 hour with increasing doses of deguelin (Deg; 0–100 μM), and co-cultured in the presence or absence of the murine *Ltk*^*-*^ feeder cell line and fludarabine (Flu; 0–50 μM). After 48h, apoptosis was determined by Annexin V/PI staining and flow cytometry. (A) ED50 was calculated for each sample. Horizontal lines represent the median. * P < 0.05 (Wilcoxon matched paired test). (B) Deguelin and fludarabine were combined at a constant 1:2 molar ratio over the same range of concentrations as in (A), and the combination index (CI) values were computed at several affected fractions (ED50, ED75, ED90). Mean values and SD are shown, CI < 1 indicate synergy.

Pro-survival signals provided by the tissue microenvironment can be mimicked *in vitro* by co-culture of CLL cells with stromal cells [42]. To study the ability of deguelin to induce apoptosis in CLL cells cultured under pro-survival conditions, PBMCs from 8 CLL patients were treated with increasing doses of deguelin (0–100 μM) or fludarabine (0–50 μM) for 48h, alone or with *Ltk*^-^ cells. As previously described [43], co-culture of CLL cells with *Ltk*^*-*^ significantly reduced their spontaneous apoptosis from 33.1 ± 13.3% to 8.8 ± 5.8% (P < 0.0005). Deguelin showed similar efficacy against CLL cells cultured alone or over *Ltk*^*-*^ cells, whereas the ED50 for fludarabine was increased about 6-fold in the presence of *Ltk*^*-*^ cells (Fig 3A).

We then examined the effect of the combination of deguelin and fludarabine. For this, both drugs were combined at a constant 1:2 molar ratio over the range of concentrations shown above and tested in 8 CLL samples. After 48 h, efficacy of the combination was measured by calculation of the combination index (CI) values from dose-response profiles at several affected fractions (ED50, ED75, ED90, see methods). The results indicate a moderate synergy which was similar in the absence or presence of *Ltk*^*-*^ in culture (Fig 3B), and led to dose reduction index (DRI) values for fludarabine of 2–3 in both culture conditions.

### Deguelin downregulates AKT/NFκB mediated prosurvival signals in CLL cells

**Fig 4:**
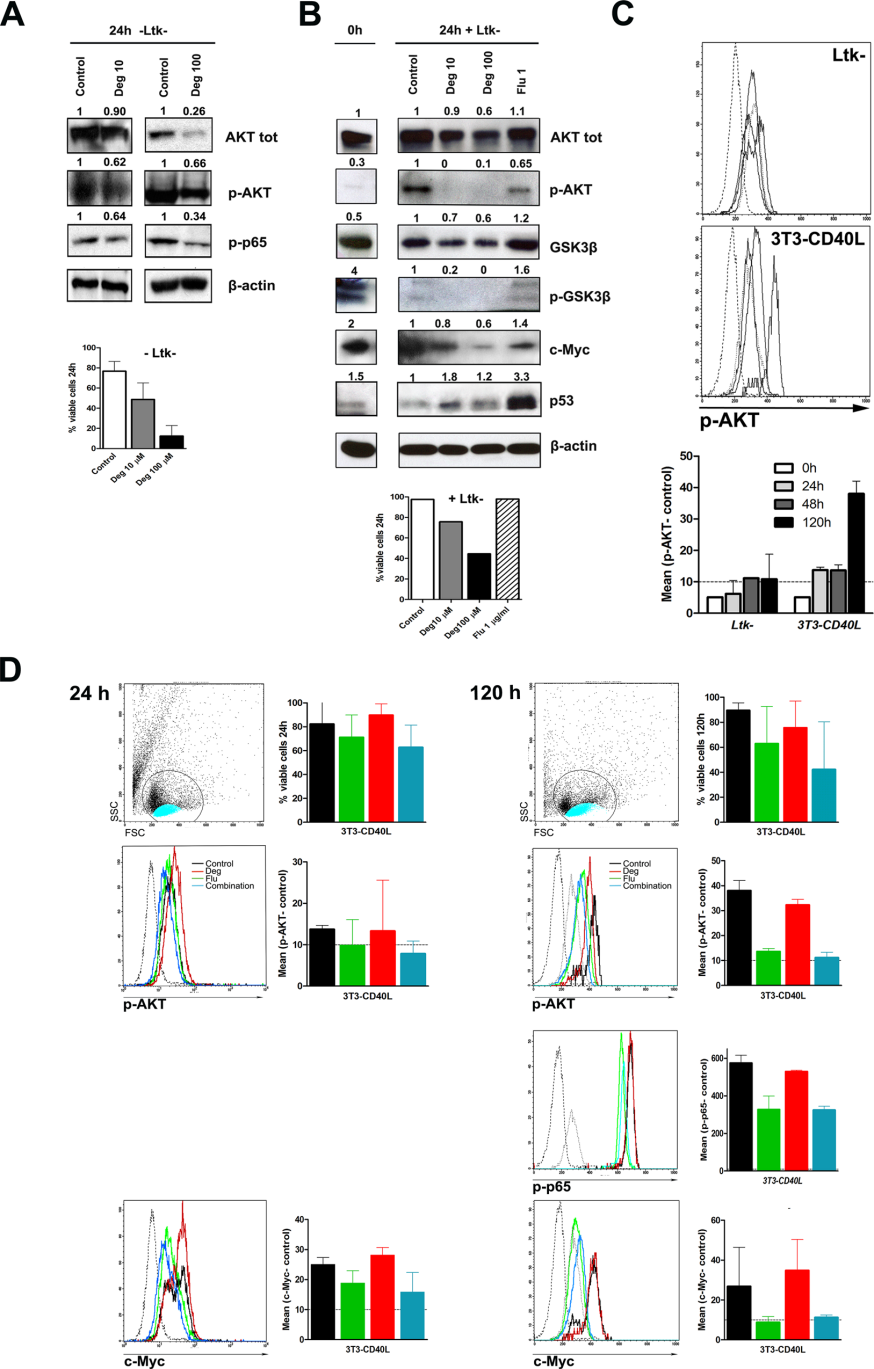
Deguelin downregulates AKT/NFκB mediated prosurvival signals in CLL cells. (A) PBMCs from 3 CLL patients were treated for 24h with 0, 10, or 100 μM deguelin. Viability was evaluated by flow cytometry after Annexin V/IP staining. Figure shows immunoreactive bands for several deguelin target molecules and β-actin in western blots from a representative sample. Numbers indicate the signal intensity of β-actin-normalized bands from drug treated samples compared to the untreated ones (Control). (B) PBMCs from two CLL patients were treated with 0, 10, or 100 μM deguelin or 1 μg/ml fludarabine and co-cultured with *Ltk*^*-*^ 24h. Cell viability assays and western blot from a representative sample were done as in (A). (C) PBMCs from 2 CLL patients were co-cultured with *Ltk*^*-*^ or 3T3-CD40L cells and p-AKT expression was evaluated in CLL cells by intracellular staining and flow cytometry at different time points. Histograms show the stains in one of the samples (solid lines from left to right: p-AKT staining at 0, 24, 48 and 120h; dotted lines from left to right: unstained control (samples without primary antibody plus secondary antibody) and isotype control. The lower graph shows the differences in the Mean Fluorescence Intensity (MFI, in channel numbers) of p-AKT compared to the unstained control in both samples (bars show mean and SD). The dotted horizontal line indicates the mean difference in MFI (10 ± 5) between the isotype controls and corresponding unstained controls in the samples with both stains (n = 8), that represents an estimation of the limit for a clearly specific fluorescence signal. (D) PBMCs from 2 CLL patients were treated with 10 μM deguelin, 1 μg/ml fludarabine or the combination of both, and co-cultured with the 3T3-CD40L cell line up to 120h. Upper graphs show viability of CLL cells in both samples (black: control; green: deguelin; red: fludarabine; blue: deguelin plus fludarabine; dotted lines from left to right: unstained control and isotype control). FSC/SSC plots from samples treated with the combination of both drugs are also shown. Histograms show the expression of p-AKT, p-p65 and c-Myc in a representative sample evaluated by intracellular staining and flow cytometry and lower graphs show MFI differences in both samples as in (C).

The AKT/NFκB pathway is central to CLL cell survival and acquisition of resistance to fludarabine-based cytotoxic therapy [9,43]. To test AKT/NFκB inhibition by deguelin, PBMCs from 3 CLL patients were treated *in vitro* with 10 μM and 100 μM deguelin for 24 h. Consistent with previous reports in CLL and other tumor cells [23,26], deguelin downregulated the expression of AKT and the activity of NFκB (p-p65) (Fig 4A). This result, however, does not necessarily imply that deguelin induces apoptosis in CLL cells by downregulating AKT/NFκB activity. Because there are significant differences in the proportion of apoptotic cells between control and deguelin treated cells, if levels of p-AKT, NFκB and antiapoptotic proteins were low in apoptotic cells regardless the cause of cell death, that same result would be obtained irrespective of deguelin-induced apoptosis being mediated by inhibition of AKT/NFκB or not. In order to get a more direct evidence about the action of deguelin on the AKT/NFκB pathway in CLL cells we treated 2 additional CLL samples with deguelin, fludarabine or both, alone or in the presence of *Ltk*^-^ or 3T3-CD40LG. Total AKT, p-AKT, and several other proteins (GSK3β, p-GSK3β, c-Myc, p53) were measured in WB in 24h deguelin or fludarabine treated CLL cells cultured with *Ltk*^*-*^. Apoptosis of CLL cells with *Ltk*^-^ was significantly lower than in cells cultured alone, so the possible confounding by different proportions of apoptotic cells between treatments would be reduced in this case (with the exception of the 100 μM deguelin condition). Results of this WB show how coculture with *Ltk*^*-*^ induces p-AKT expression in CLL cells and deguelin reduced total AKT and p-AKT levels (Fig 4B). Levels of the AKT target GSK3β were also reduced by deguelin, and p-GSK3β (lower band with the p-GSKαβ Ser^21,9^ antibody used) was undetectable already with 10 μM deguelin. Fludarabine did not reduce total AKT levels, but decreased p-AKT levels compared to control cells. Of note, however, reduction of p-AKT seen with fludarabine was not accompanied by reduced p-GSK3β, and was not replicated in the results shown below. In agreement with its known DNA damage inducing effect, fludarabine increased p53 expression notably, whereas only a minor increase over control cells was seen in deguelin treated cells (Fig 4B).

Additionally, p-AKT and p-p65 were also measured by intracellular staining and flow cytometry. The good and reproducible correlation of live and apoptotic cells with their position in FSC/SSC plots (S3 Fig) allowed us to measure protein levels in the live and apoptotic fraction separately. Apoptotic cells were indeed shown to have lower expression of all the proteins measured, regardless of treatment or time point (S3 Fig). In the live fraction of cells, p-AKT gave only fluorescence well above the isotype controls at the longer culture time point of 120h (Fig 4C). Nevertheless, overall results were similar for 24h and 120h, showing that deguelin, but not fludarabine, downregulated p-AKT expression in CLL cells (Fig 4D). The combination of deguelin plus fludarabine did not result in greater downregulation compared to deguelin alone. Expression of p-p65 followed that of p-AKT, again with deguelin and deguelin plus fludarabine reducing p-p65 levels to a similar extent, and no effect of fludarabine alone (Fig 4D.

Recent findings have revealed an important role for c-Myc in microenvironment mediated CLL cell survival and drug resistance [11,44]. In WB of 24h treated CLL cells cultured with *Ltk*^*-*^ cells, c-Myc expression was downregulated by deguelin in a dose-dependent manner. Expresion of c-Myc was also measured by flow cytometry, and paralleled that of p-AKT in these experiments, with a good signal being clearly detectable only in CLL cells cultured with 3T3-CD40LG cells, in this case already at early time points (Fig 4D). Similar to p-AKT, and in agreement with the result in WB, c-Myc was downregulated by deguelin but not by fludarabine, with the combination showing no further effect (Fig 4B and 4D).

Taken together, this results show that downregulation of p-AKT and p-p65 by deguelin takes place in the live fraction of cells, where only a minority of cells stain positive forAnnexin V (S3 Fig), indicating that downregulation of AKT/NFκB by deguelin precedes the onset of apoptosis, and thus strongly suggesting that deguelin induces apoptosis in CLL cells by downregulation of the AKT/NFκB pathway.

### Potentiation of fludarabine action by deguelin as possible mechanism of synergy

**Fig 5:**
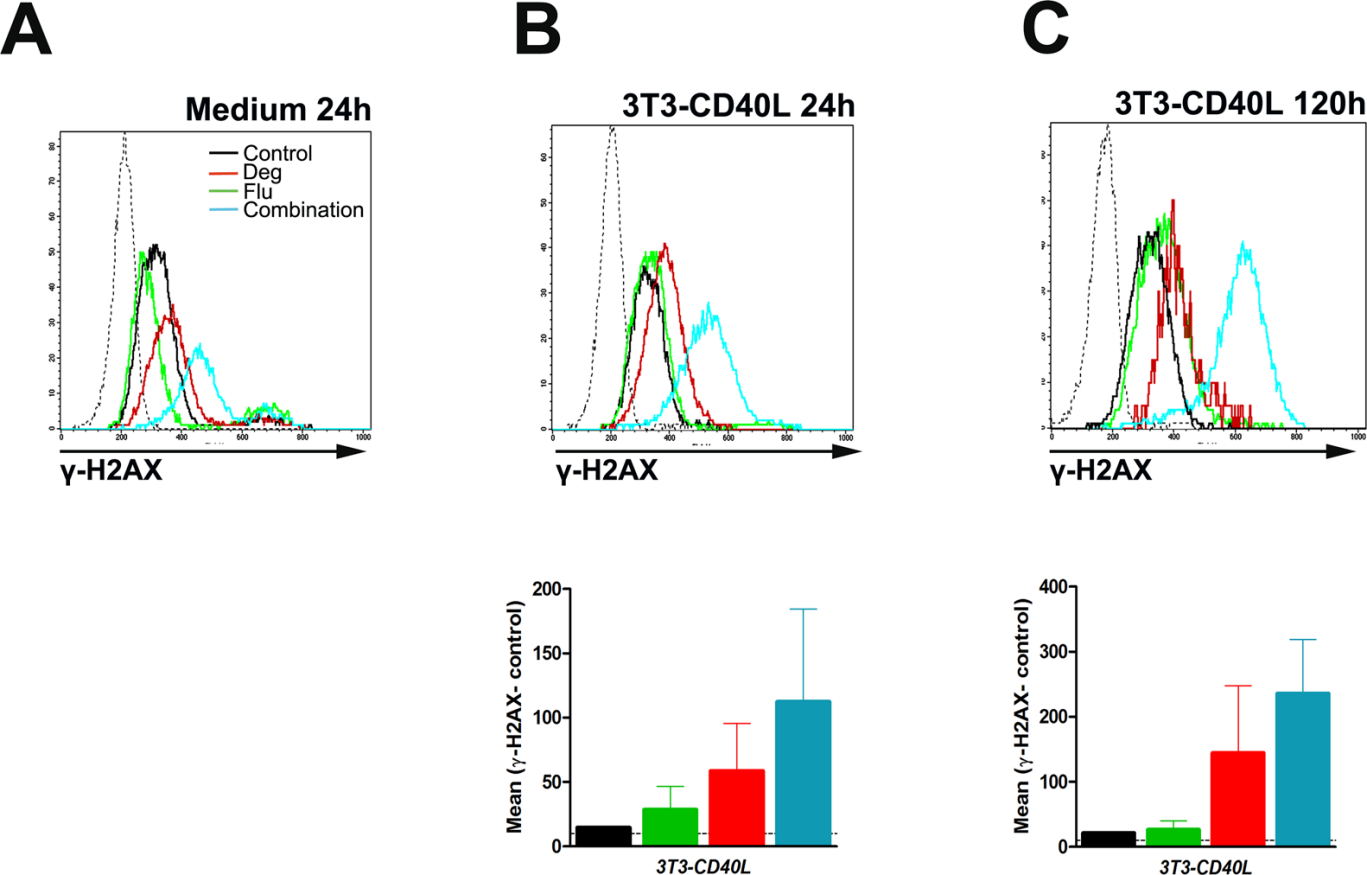
Potentiation of fludarabine action by deguelin. PBMCs from 2 CLL patients were treated with 10 μM deguelin, 1 μg/ml fludarabine or the combination of both, and co-cultured with 3T3-CD40L cell line up to 120h (same samples as in Fig 4B, 4C and 4D). Histograms show the expression of γ-H2AX in a representative sample evaluated by intracellular staining and flow cytometry (black: control; green: deguelin; red: fludarabine; blue: deguelin plus fludarabine; dotted lines from left to right: unstained control and isotype control (see Fig 4C)). The lower graphs shows the differences in the Mean Fluorescence Intensity (MFI) of γ-H2AX staining compared to the unstained control in both samples (bars show mean and SD). The dotted horizontal lines indicate the mean difference in MFI between the isotype controls and corresponding unstained controls (see Fig 4C).

Several Hsp90 inhibitors have been recently shown to act synergistically with fludarabine on CLL cells [45,46] by increasing fludarabine-induced DNA damage [47]. Because the combination of deguelin and fludarabine had also been found to be synergistic, we tested if deguelin was able to increase the efficacy of fludarabine on CLL cells. Fig 5 shows intracellular stains of phosphohistone γ-H2AX as a marker of dsDNA strand breaks. As expected, fludarabine caused DNA damage in CLL cells. The increase in γ-H2AX fluorescence was already seen in the live fraction of cells already at 24h, and similar in CLL cells cultured alone or with 3T3-CD40LG (Fig 5A and 5B), but was more pronounced at 120h (Fig 5C). Contrary to fudarabine, deguelin did not increase γ-H2AX expression, what is in agreement with p53 expression (Fig 4A). However, the combination of deguelin plus fludarabine produced a remarkable increase in γ-H2AX fluorescence compared to fludarabine alone (Fig 5B and 5C). This suggests that potentiation of fludarabine action by deguelin may be largely responsible for the synergy they show against CLL cells.

### Deguelin induces apoptosis in NZB CLL-like cells

**Fig 6:**
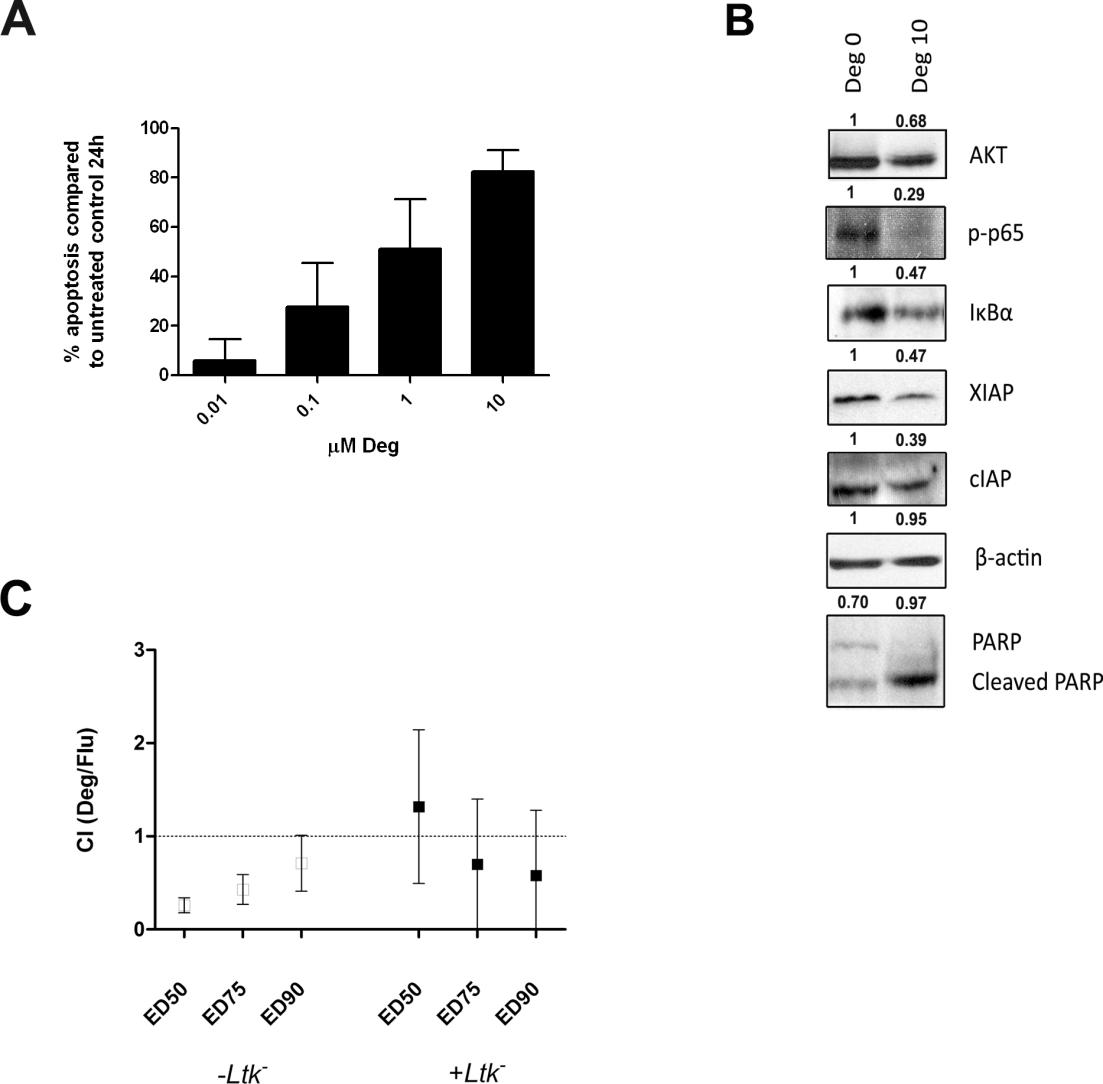
Deguelin induces apoptosis in NZB CLL-like cells. (A) Mononuclear cells isolated from 8 NZB spleens were cultured in the presence of deguelin (Deg; 0–10 μM) and apoptosis was quantified at 24h. (B) Cell lysates obtained from splenic NZB cells treated with 10 μM deguelin for 24h were analyzed by western blot. Immunoreactive bands for several deguelin target molecules from a representative sample are shown. Numbers indicate the signal intensity of β-actin-normalized bands from deguelin treated samples (Deg 10) compared to the untreated ones (Deg 0). For PARP, numbers indicate the proportion of cleaved fragments relative to total protein (intact plus cleaved). (C) Splenic mononuclear cells from 8 NZB mice were treated with increasing doses of deguelin (0–100 μM), fludarabine (0–50 μM) or combinations of both (at a constant 1:2 molar ratio) and co-cultured with or without *Ltk*^*-*^. After 48h, the combination index (CI) values were computed at several affected fractions (ED50, ED75, ED90). Mean values and SD are shown. CI < 1 indicate synergy.

We then tested *in vitro* the effect of deguelin on CLL-like cells from the spleens of 4 aged NZB mice and 4 transplanted NZB mice (all samples had ≥ 90% B220^low^ CD5^low^ IgM^+^ leukemic B cells, as assessed by flow cytometry). Similar to the effect in human CLL cells, deguelin induced apoptosis in NZB CLL-like cells in a dose-dependent manner (Fig 6A), and led to downregulation of AKT, NFκB (p65) activity, IκBα and the antiapoptotic proteins cIAP and XIAP (Fig 6B). Deguelin was more effective against NZB CLL-like cells than towards human CLL cells in this assays (median ED50 at 24h: 1.1 μM vs 6.2 μM, respectively, p = 0.0006, Mann-Whitney U test). Again, we tested *in vitro* the effect of the combination of deguelin and fludarabine in these cells, and they showed mild synergy in a similar way to that found in human CLL cells (Fig 6C).

### Effect of deguelin in NZB CLL-like cells *in vivo*

**Fig 7:**
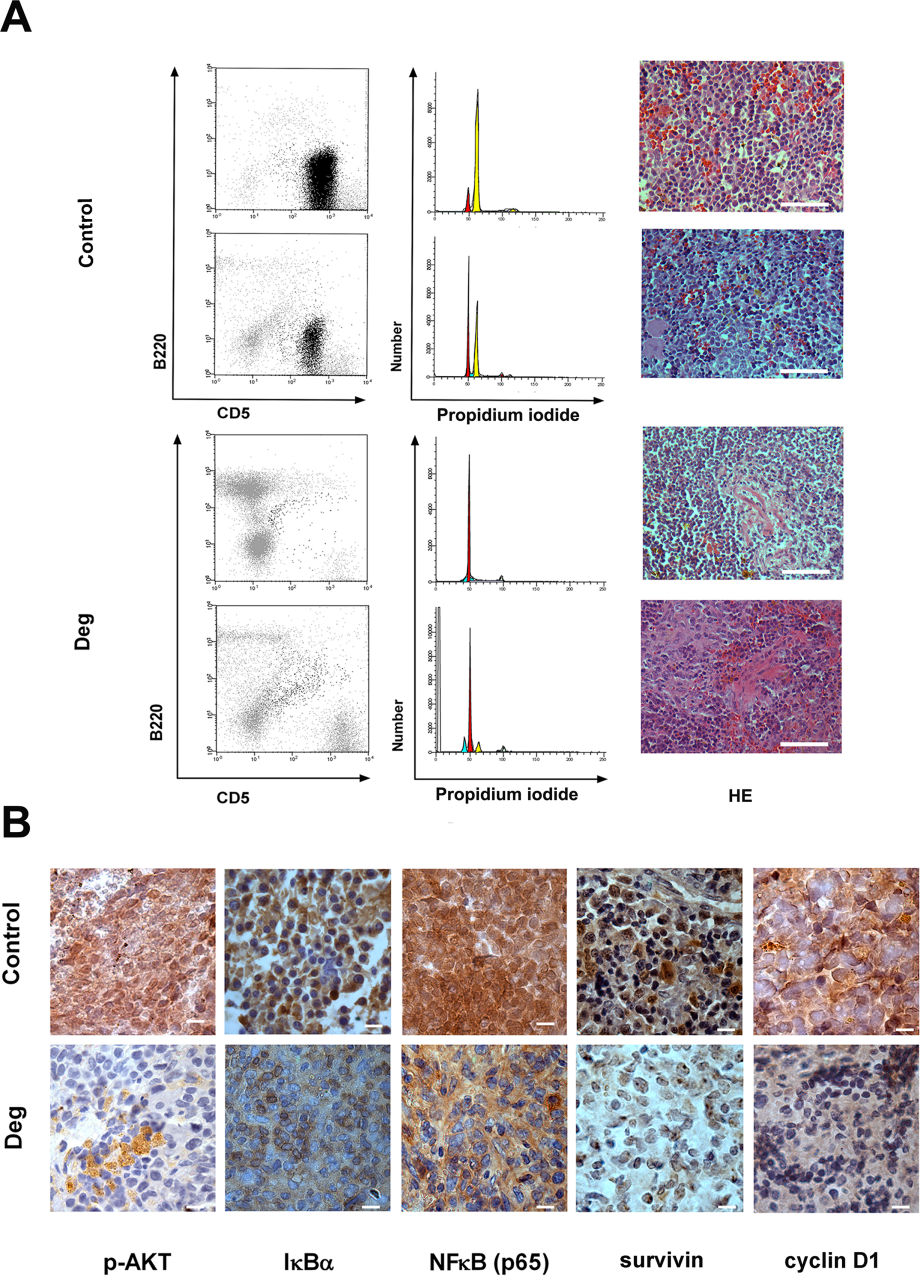
Effect of deguelin *in vivo* in transplanted NZB mice. Five transplanted animals with splenomegaly were selected to study the effect of deguelin *in vivo*. Two mice received deguelin (4 mg/kg in corn oil) twice a day for three consecutive days by i.g. route and the other three received vehicle. Mice were then sacrificed, spleens were excised and samples for mononuclear cell isolation and histological analysis were obtained. (A) Flow-cytometric analysis of NZB spleen cells (left panels), with neoplasic B220^low^ CD5^low^ IgM^+^ cells represented by black dots. Analysis of DNA content from NZB splenic cells are shown in middle panels. Blue peaks represent apoptotic cells, red ones normal diploid cells and yellow ones hyperdiploid neoplastic cells (peaks shown are computer modeled). Histological analysis of spleens stained with hematoxylin and eosin (HE) are shown in right panels (× 40 magnification, scale bar 50 μm). Data from two untreated animals (Control) and two treated animals (Deg) are shown. (B) Paraffin embedded spleen sections from untreated (Control) and deguelin-treated (Deg) animals were processed for immunohistochemical staining of the indicated protein. Brown = specific protein staining, Blue = counterstaining of nuclei with hematoxylin. Representative images were taken at 100 x augments (scale bar 10 μm).

To examine the action of deguelin *in vivo*, 5 young mice were transplanted with spleen cells from one transplanted mice. Six months following transfer splenomegaly was evident upon palpation in all mice. Two of them were treated with deguelin (4 mg/kg) twice a day for three consecutive days, the other three received vehicle alone. On the fourth day all animals were sacrificed, their spleens were excised, samples for immunohistochemistry were obtained and mononuclear cells were isolated. Flow-cytometry analysis showed a striking decrease in the number of leukemic hyperdiploid B220^low^ CD5^low^ IgM^+^ cells in deguelin-treated animals compared to controls (Fig 7A, left panels). Cell cycle analysis of spleen cells after DNA staining with PI showed the almost complete disappearance of the hyperdiploid leukemic cells in deguelin treated spleens (Fig 7A, middle panels). Microscopic examination revealed that control spleens contained dense viable tumor cells, in contrast to the large necrotic and fibrotic areas observed in tumors from the deguelin-treated group (Fig 7A, right panels). Accordingly, immunohistochemical analysis of spleens from deguelin-treated mice revealed a marked reduction in p-AKT, IκBα and nuclear NFκB p65 staining, in sharp contrast with spleens from control mice (Fig 7B). Also, the immunohistochemical staining of two NFκB-regulated proteins, survivin and cyclin D1 was markedly reduced in deguelin treated mice (Fig 7B).

### Combined treatment with deguelin and fludarabine prolongs the survival of transplanted NZB mice

To explore the therapeutic potential for deguelin as adjuvant for fludarabine-based therapy, 40 transplanted mice were randomly distributed in four groups. Tumor engraftment was confirmed two months later (age 12–13 weeks) by detection of leukemic cells in the peripheral blood. By that moment, all transplanted mice had detectable hyperdiploid leukemic cells in the peripheral blood (in contrast to three 6 month old non-transplanted NZB mice, S1 Fig), indicating that the leukemic cells detected most likely derive from the transplanted spleen cells. One month later, treatment was initiated and they received vehicle or deguelin (4 mg/kg) three days per week for 61 days, plus vehicle or fludarabine (35 mg/kg) five consecutive days for three cycles starting each 28 days. After the treatment period, mice were monitored and survival was compared between groups by Kaplan-Meyer analysis. Animals (n = 9) that died before the completion of the treatment period were censored. Five of them that were found dead were not autopsied and the cause of death is unknown. Another one died due to a sudden cardiac arrest during routine manipulation. The other three were sacrificed for meeting end point criteria. Autopsies revealed glomerulonephritis in one of them, and CLL-like lymhoproliferation in the other two. All other mice were included in the survival study and died as a consequence of CLL-like disease. There was no evidence of significant toxicity in any of the treatment groups, as seen by the weights (S2 Fig) and behavior of the mice included in this experiment.

**Fig 8:**
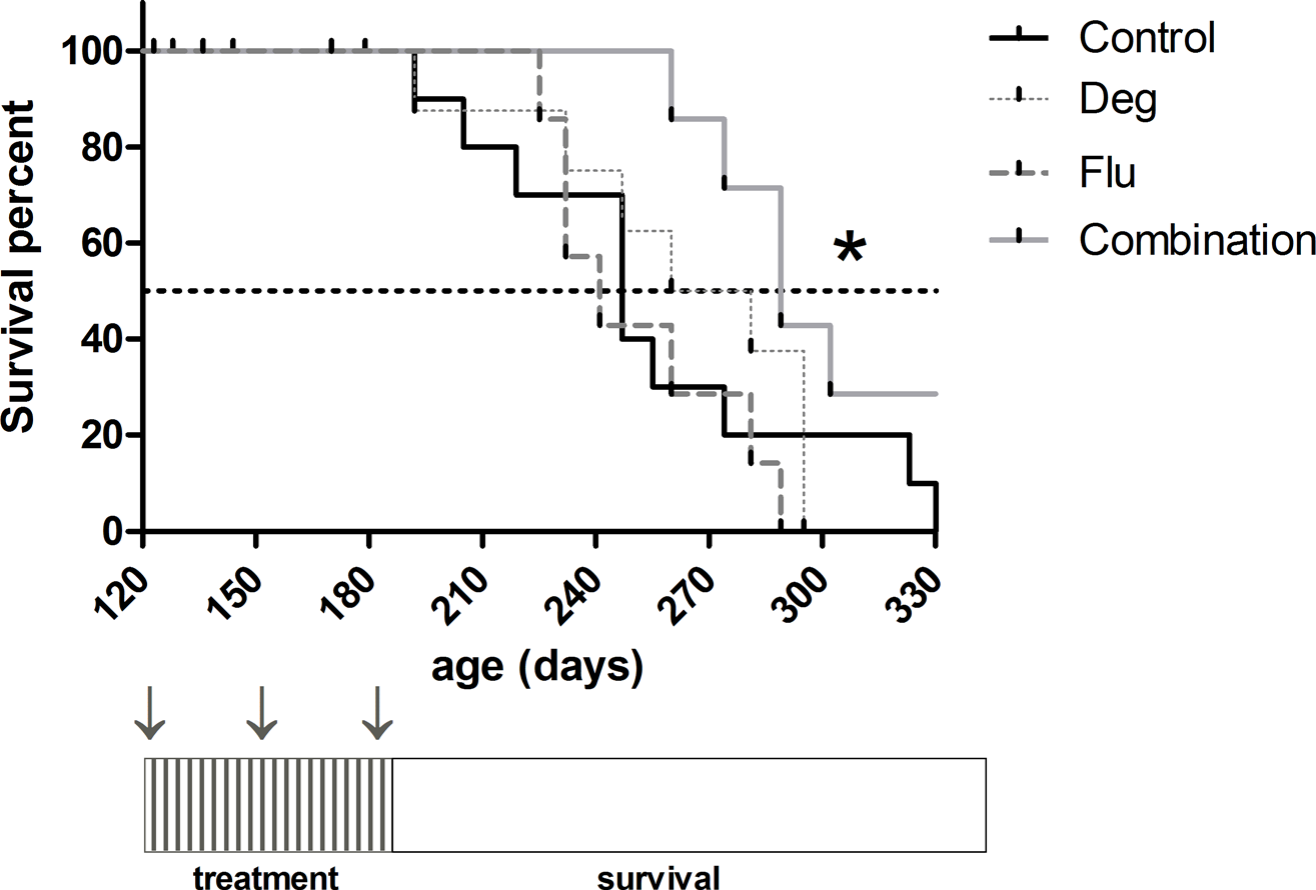
The combination of deguelin and fludarabine prolongs the survival of transplanted NZB mice. 40 transplanted young NZB mice were randomly distributed in four groups and transplanted with leukemic spleen cells from aged NZB. Control group (Control, solid black line) received vehicle i.g. and i.p. Fludarabine (Flu) group received 3 cycles of 35 mg/kg fludarabine i.p., five consecutive days each 28 days. Deguelin group (Deg) received 4 mg/kg deguelin in corn oil i.g. three days per week for 61 days. The last group (Combination) received deguelin plus fludarabine as in groups Deg and Flu. Treatment schedule is shown in the lower diagram (Deg:**||||**; Flu: **↓**). After treatment, mice were monitored and survival was evaluated. Graph shows survival of mice in the four groups (see legend). *P = 0.022 (Gehan-Breslow-Wilcoxon test).

Statistical analysis showed that whereas deguelin and fludarabine as monotherapy did not increase survival at the doses and regimen tested, the combination of deguelin plus fludarabine increased significantly the survival of mice when compared to vehicle, deguelin alone or fludarabine alone (289, 247, 277 and 241 days respectively, P = 0.022, Fig 8). The survival curves show that deguelin plus fludarabine treated mice lived longer until deaths begun to occur with similar paucity as in the other groups. This suggests deguelin plus fludarabine administration were able to more effectively slow the progression of disease, but progression resumed soon after treatment was stopped.

## Discussion

In line with previous reports, in this work we show how deguelin causes apoptosis in CLL cells *in vitro*, with more activity towards CLL cells than healthy T lymphocytes from CLL patients or PBMCs form healthy individuals, and showing mild synergy with fludarabine. We show how deguelin downregulates AKT and NFκB in CLL cells, in agreement with previous reports on deguelin action in several cancer cell lines and also in CLL cells [25,26]. Moreover, we show how deguelin downregulation of AKT and NFκB takes place prior to the onset of apoptosis, strongly pointing to the inhibition of the AKT/FκB pathway as a fundamental mechanism of apoptosis induction in CLL cells by deguelin. The combination of deguelin with fludarabine did not increase the magnitude of AKT/NFκB downregulation seen with deguelin alone, but deguelin did remarkably increase DNA damage induced by fludarabine, suggesting that potentiation of fludarabine action by deguelin play an important part in the synergy found with the combination of both drugs *in vitro*.

There are two known cellular targets for deguelin: the mitochondrial NADH:ubiquinone oxidoreductase complex (Complex I) and the chaperone Hsp90, which is inhibited by deguelin through its binding to the N-terminal ATP binding site [21]. Hsp90 is overexpressed in most if not all cancers [48] and selectively localizes to the mitochondria of cancer cells [49]. Inhibition of Hsp90 results in downregulation of its client proteins [50], among them several oncogenic kinases like AKT, Raf-1, v-src [51–53] and also some important proteins in the biology of CLL cells like ZAP-70 [54], BCL2 and survivin [51]. Inhibition of Hsp90 has been explored in the last years for the potential treatment of a variety of cancers [50,55,56], including CLL, where the Hsp90 inhibitors NVP-AUY922-AG and SNX-7081 have been shown to inhibit AKT and NFκB, and to act synergistically with fludarabine [45,46,57].

The effects of deguelin on CLL cells shown in this work agree with the mechanisms of action described for this molecule. Deguelin downregulates AKT and several downstream targets along the AKT/NFκB pathway that are important for CLL cell survival, like p-65 NFκB subunit, GSK3β, c-Myc, cIAP1/2, XIAP or survivin. Some of them are also Hsp90 clients, like GSK3β, Cyclin D1, Survivin or BCL2 [51,58], so Hsp90 inhibition and subsequent AKT downregulation are likely to reinforce each other at several points along the AKT/NFκB pathway.

We also show how deguelin, but not fludarabine, is able to inhibit stromal cell-induced c-Myc expression. Inhibition of c-Myc by deguelin in CLL cells shown in this work is an interesting finding in light of its recently described relevance in the metabolic changes that take place in CLL cells upon contact with their tissue microenvironment, changes that promote CLL cell survival and resistance to chemotherapeutic drugs [59]. This finding is in agreement with already described inhibition of c-Myc by Hsp90 inhibitors [60,61], and could explain the synergy found between deguelin and fludarabine. Postulated mechanisms explaining this synergy between Hsp90 inhibitors and fludarabine include downregulation of antiapoptotic proteins as a consequence of AKT/NFκB inhibition [45] or downregulation of DNA repair enzymes and checkpoint regulators that limit the capacity of CLL cells to repair fludarabine-induced DNA damage [61]. According to our results, both AKT/NFκB inhibition and reinforcement of fludarabine-induced DNA damage take place in degluelin plus fludarabine treated CLL cells, and likely cooperate in induction of apoptosis. Potentiation of fludarabine action by deguelin could be largely mediated by inhibition of the increase in cellular ATP content induced by c-Myc. Raised ATP levels have been shown to render colon cancer cells resistant to alkylating agents [62]. The efficacy of fludarabine in inducing dsDNA strand breaks has been shown to be proportional to the ratio of fludarabine/dATP [63], and resistance to fludarabine in CLL has been shown to correlate with increased ATP levels induced by contact with stromal cells [59], so lowering ATP levels as a consequence of c-Myc inhibition is likely to result in heightened fludarabine effectiveness.

Given the efficacy of deguelin and the combination with fludarabine against CLL cells found *in vitro*, we decided to test the potential of these drugs for CLL treatment in the NZB mouse strain. Similar to human CLL cells, deguelin induced apoptosis in cultured CLL-like cells from NZB mice by inhibiting the activity of AKT and NFκB, leading to downregulation of antiapoptotic proteins. The spontaneous lymphoproliferation in NZB mice represents a model of human CLL, but have several drawbacks for its use in testing potential therapeutic compounds. Lymphoproliferation develops at an advanced age, and only in a fraction of mice. Besides, this strain spontaneously develop autoantibodies and autoimmunity that resembles autoimmune hemolytic anemia and systemic lupus erythematosus, a phenomenom that represents a similarity between the NZB strain and human CLL [64,65], but causes a significant fraction of wild type NZB mice to die as a consequence of autoimmunity and not lymphoproliferation. By transplanting spleen cells from aged NZB mice with leukemic CLL-like cells into young NZB recipients, lymphoproliferation presented with virtually complete penetrance, and disease onset was anticipated compared to wild-type NZB mice, with all mice showing leukemic cells in the peripheral blood by 12 weeks of life, well before their spontaneous appearance in wild-type NZB mice [27]. Autopsies of transplanted control mice revealed, besides massive splenomegaly in all the animals, a majority of cases with infiltration of tumor cells in the bone marrow, approximately half with infiltration in the liver and a quarter with infiltration of mesenteric lymph nodes. Although all mice were Coombs positive at the time of sacrifice, and all had IgG anti-dsDNA (not shown), glomerulonephritis was found only in one mice (one of the censored mice in the survival study), and the life span of the transplanted mice was significantly shortened compared to wild type NZB mice, with all control mice dead before 11 months of life, when 85% of wild type NZB mice are still alive [66], indicating that the transplants lead to an anticipated leukemia that became the main cause of death, thus making it a model better suited for preclinical studies.

Deguelin given orally at 4 mg/kg twice a day for only 3 days showed a remarkable activity against transplanted NZB CLL-like cells, leading to the almost complete disappearance of hyperdiploid leukemic cells from the spleens of diseased mice. However, caution must be taken with this result, as it has been obtained in a small number of animals and we have no direct measure of the numbers of leukemic cells before and after deguelin action in the treated animals. Nevertheless, the fact that the five mice used for this assay were transplanted with spleen cells from the same diseased mice and all, control and deguelin treated, had a similar degree of splenomegaly with the 3 control mice bearing a prominent population of CLL-like cells, altogether support the idea of a notable effect of deguelin against the leukemic cells in this experiment. Other inhibitors of kinases affecting the AKT/NFκB pathway, like the PI3Kδ inhibitor Idelalisib and the BTK inhibitor Ibrutinib, have recently shown a potent effect in disturbing the homing of CLL cells, each by different mechanisms [67,68], causing their mobilization from secondary lymphoid organs. The rapid disappearance of the majority of leukemic hyperdiploid cells in the 3 day deguelin treated mice could reflect a similar effect of deguelin on the CLL-like cells residing in the spleen. Unfortunately, we do not have leukemic cell counts in blood samples from the five mice included in that experiment prior to deguelin treatment, and only in the two deguelin treated and one control mice after the 3 day deguelin treatment. In those three mice the percentage of leukemic hyperdiploid cells in the peripheral blood was similar, and examination of spleens from deguelin treated mice revealed extensive fibrotic areas of healing tissue, suggesting that CLL-like cells in the spleens of treated mice underwent apoptosis rather than mobilization to the peripheral blood.

In another experiment aimed to test *in vivo* the potential benefit of the interaction of deguelin with fludarabine, both compounds in combination prolonged the survival of treated mice compared to controls, deguelin or fludarabine alone, indicating that the addition of deguelin to ineffective doses of fludarabine resulted in an effective combination, what is in line with the DRI of 2–3 in the dose of fludarabine obtained with the addition of deguelin *in vitro*. These results suggest a potential role for the combination of deguelin with fludarabine as therapy for CLL. The fact that deguelin alone did not prolong the survival of mice compared to controls in the survival study contrasts with the high activity seen in the previous experiment with 8 mg/kg for just 3 days. Regarding this, notable differences have already been described in the effectiveness of deguelin resulting from narrow differences in the doses administered, as in a carcinogen-induced model of colon cancer [15], where 5 mg/kg deguelin daily for 6 weeks reduced by four-fold the number of carcinogen-induced aberrant crypt foci compared to controls, whereas 2.5 mg/kg did not have a significant effect. This suggest that the 4 mg/kg, 3 days per week schedule we used for the survival study was below the minimal effective dose against the NZB CLL-like cells, but 4mg/kg twice a day reached and maintained an effective concentration in the tissues of mice. This underscores the importance of finding the optimal dose and regimen of administration in order to obtain the maximum benefit and suggests that, in the case of deguelin, higher doses given in a shorter time period could be more effective than lower doses given over longer time periods, resulting in a lower total accumulated dose of deguelin. Several preclinical studies have reported that deguelin can be administered orally at doses of up to 8 mg/kg in mice twice daily for at least 28 days without significant toxicity, [15,18,21,69], what indicates that the dose of 8 mg/kg per day for three days we used in our assay is tolerable, and could be safely maintained for longer time periods. Our results support previous findings on the activity of deguelin against CLL cells, further show that effective doses against CLL cells can be achieved *in vivo*, and altogether warrant further investigation to determine the potential of deguelin for the treatment of CLL.

## Supporting Information

S1 Fig Engraftment confirmation in transplanted mice included in the survival study. 40 healthy mice (4 weeks old) were transplanted with leukemic spleen cells pooled from 3 aged NZB mice (TX in Fig). Mice were randomly distributed in four groups of treatment: control, deguelin (Deg), fludarabine (Flu) and deguelin plus fludarabine (Deg+Flu). Two months later blood samples were collected from and the presence of leukemic CLL-like cells was checked by flow cytometry. Bars represent the percentage of normal (B220^hi^) and leukemic (B220^low^CD5^low^IgM^+^) B cells relative to total PBMCs. No leukemic cells were detected in the peripheral blood of three non transplanted 6 month old NZB mice (no TX).
(TIF)

S2 Fig Body weight of mice included in the survival study. Animal weight values obtained before treatment (pre) and when treatment finished (post). Horizontal lines represent the mean.
(TIF)

S3 Fig Correlation of live and apoptotic cells in FCS/SSC plots allows gating of live cells in intracellular staining tubes. (A) Correlation between Annexin/IP staining and live/apoptotic cells in Ann/IP tubes. Live and apoptotic cells locate in different regions in FSC/SSC plots. Plots show two examples in 24h control and Deg (10 μM) + Flu (1 μg/ml) treated cells. Three gates were done in Ann/PI plots for live (Ann^-^/PI^-^, light blue), early apoptotic (Ann^+^/PI^-^, dark blue) and late apoptotic cells (Ann^+^/PI^+^, black). Left panels show the different location of gated cells in FSC/SSC plots. (B) Live cells can also be gated out in FSC/SSC plots from intracellular staining tubes. Two intracellular staining tubes from the same samples as in (A) were stained with phalloidin-AlexaFluor^488^, and the live cells gated out from histograms of phalloidin fluorescence (right panels, brighter peaks delimited by LR regions). Similar to Ann/PI tubes, live cells located in a defined region in FSC/SSC plots (blue cells in left panels), and the percentages of cells in the live cell region correlates well with the corresponding live cell region in Ann/PI tubes (compare percentages in LR regions of histograms in (B) with percentages in lower-left plots in (A). (C) The good correlation of live cell regions between Ann/PI and intracellular staining tubes is reproducible. CLL cells were treated with 10 μM deguelin, 1μg/ml fludarabine or the combination of both and cultured 24h with *Ltk*^*-*^ and 3T3-CD40LG cells. Graphs show the correlation between percentage of Ann^-^/IP^-^ cells and percentage of cells in the gated live region in the same Ann/PI tubes (solid symbols). The majority of gated cells in the live region were Ann^-^/IP^-^ (mean±SD: 94.5±6.1 in samples cultured with *Ltk*^*-*^ and 96.8±3.4 with 3T3-CD40LG). Replicates in the 5 intracellular staining tubes for each sample are very similar, and also have a good correlation with the percentage of cells in the live cell region in the corresponding Ann/IP tubes (compare solid and open symbols for each treatment condition). (D) Stains of p-AKT, p-p65 and c-Myc in live and apoptotic cells. Gating in the live and apoptotic cell regions in FSC/SSC plots allow seeing protein levels in live and apoptotic cells separately. Panels show cells cultured for 120h with 3T3-CD40LG cells. Plots show examples of gating in the four treatment conditions. Upper histograms show that apoptotic cells have low fluorescence in the three proteins measured, with similar intensity regardless of treatment condition. Middle panels show stains in the live fraction of cells, and lower panels in the total cell populations.
(TIF)

S1 Table CLL patient characteristics. (DOCX)
