# Supplementary figures and images for: Synergistic Activity of Deguelin and Fludarabine in Cells from Chronic Lymphocytic Leukemia Patients and in the New Zealand Black Murine Model
Source: PLoS One. 2016 Apr 21;11(4):e0154159. doi: 10.1371/journal.pone.0154159 (PMC4839760; doi:10.1371/journal.pone.0154159)

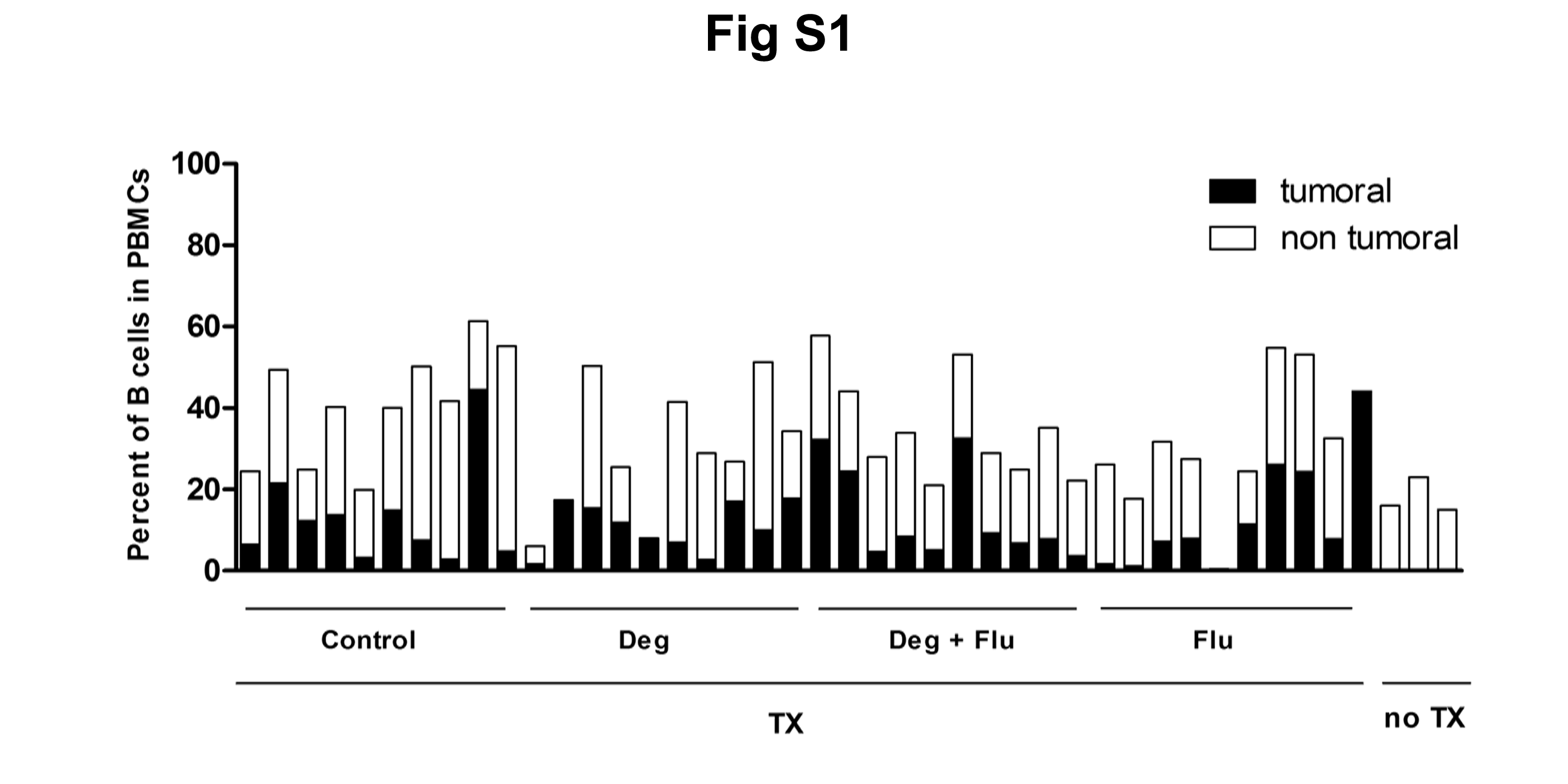

Supplement: S1 Fig — 40 healthy mice (4 weeks old) were transplanted with leukemic spleen cells pooled from 3 aged NZB mice (TX in Fig). Mice were randomly distributed in four groups of treatment: control, deguelin (Deg), fludarabine (Flu) and deguelin plus fludarabine (Deg+Flu). Two months later blood samples were collected from and the presence of leukemic CLL-like cells was checked by flow cytometry. Bars represent the percentage of normal (B220hi) and leukemic (B220lowCD5lowIgM+) B cells relative to total PBMCs. No leukemic cells were detected in the peripheral blood of three non transplanted 6 month old NZB mice (no TX). (TIF) [file pone.0154159.s001.tif]

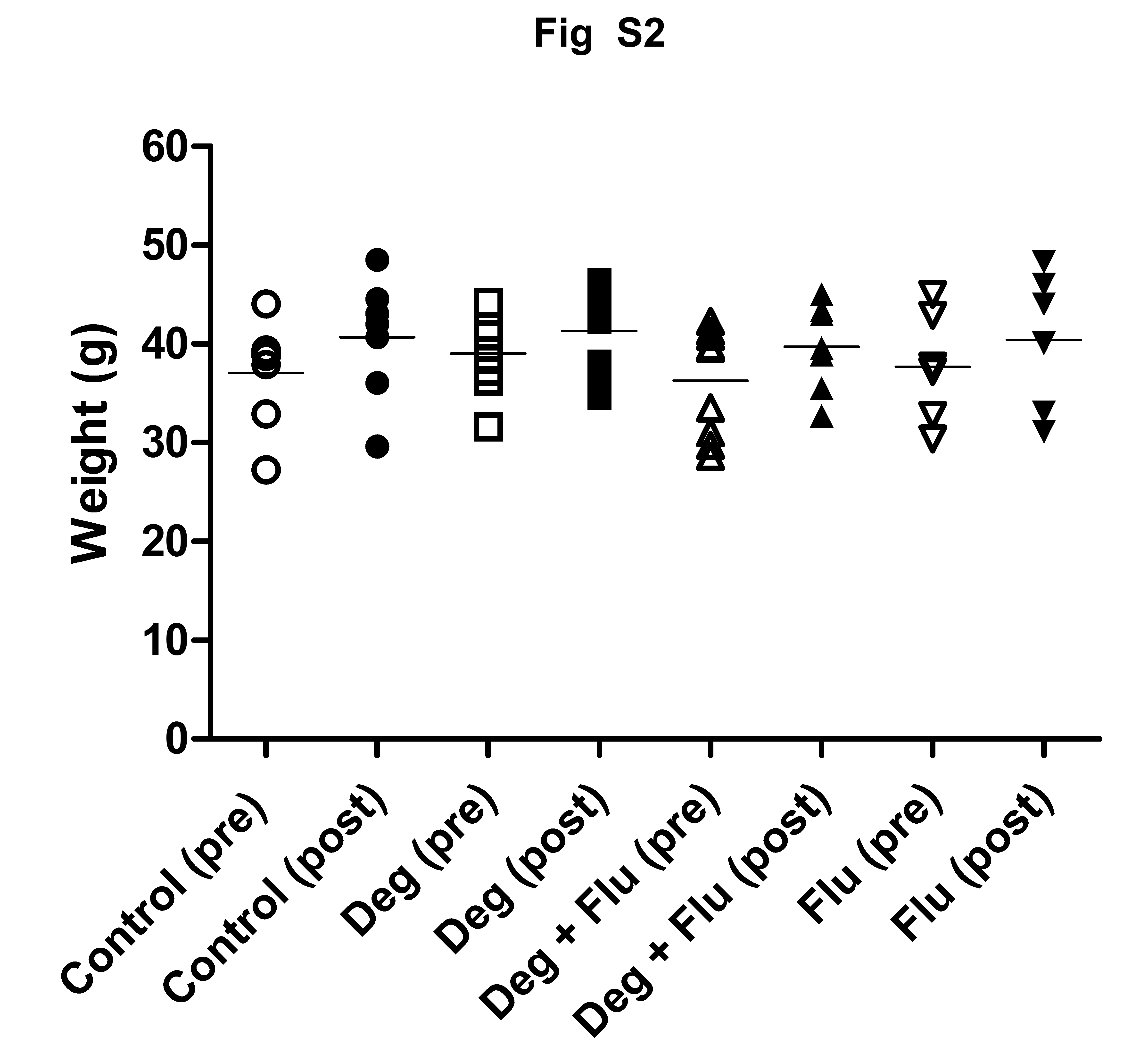

Supplement: S2 Fig — Animal weight values obtained before treatment (pre) and when treatment finished (post). Horizontal lines represent the mean. (TIF) [file pone.0154159.s002.tif]

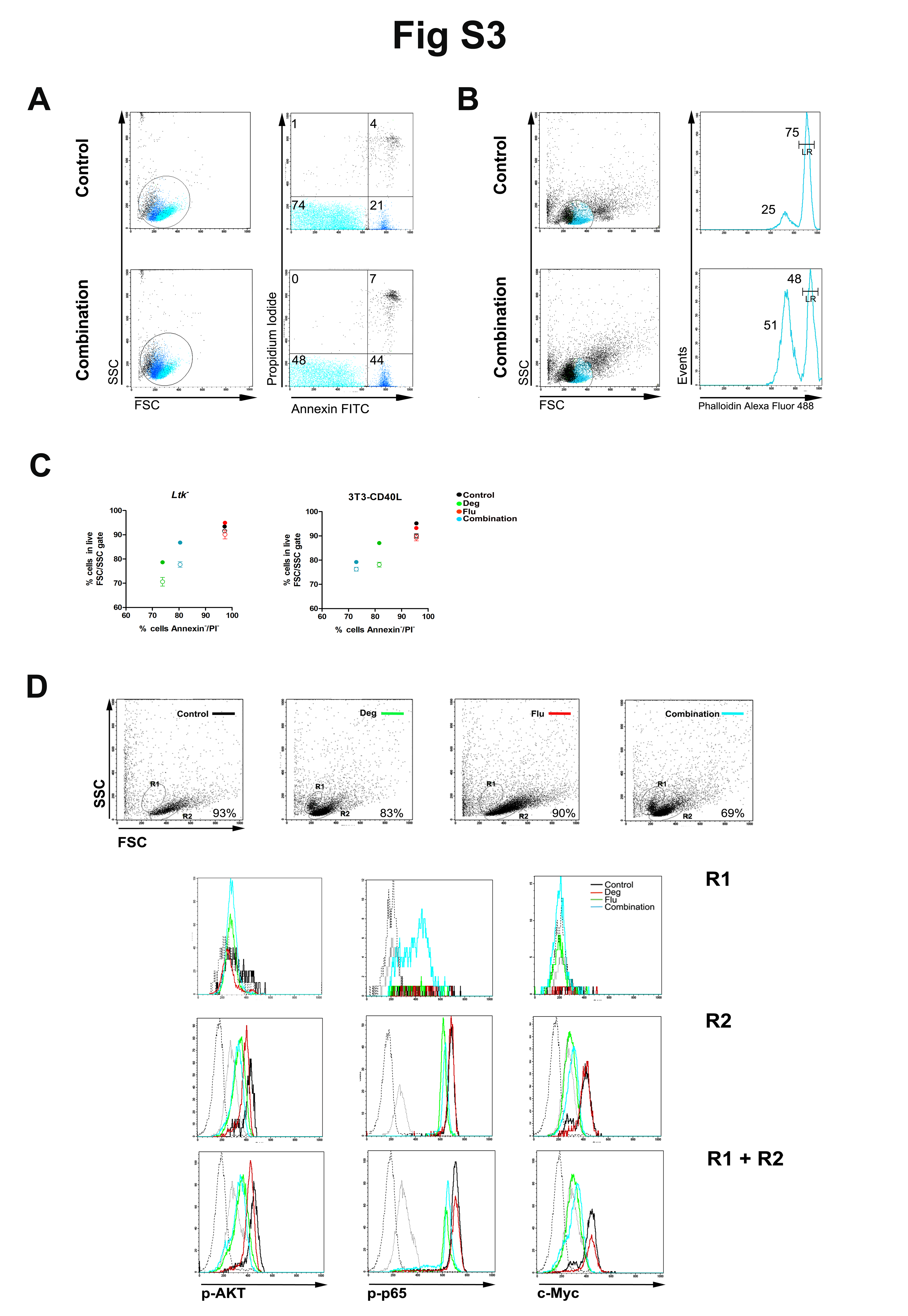

Supplement: S3 Fig — (A) Correlation between Annexin/IP staining and live/apoptotic cells in Ann/IP tubes. Live and apoptotic cells locate in different regions in FSC/SSC plots. Plots show two examples in 24h control and Deg (10 μM) + Flu (1 μg/ml) treated cells. Three gates were done in Ann/PI plots for live (Ann-/PI-, light blue), early apoptotic (Ann+/PI-, dark blue) and late apoptotic cells (Ann+/PI+, black). Left panels show the different location of gated cells in FSC/SSC plots. (B) Live cells can also be gated out in FSC/SSC plots from intracellular staining tubes. Two intracellular staining tubes from the same samples as in (A) were stained with phalloidin-AlexaFluor488, and the live cells gated out from histograms of phalloidin fluorescence (right panels, brighter peaks delimited by LR regions). Similar to Ann/PI tubes, live cells located in a defined region in FSC/SSC plots (blue cells in left panels), and the percentages of cells in the live cell region correlates well with the corresponding live cell region in Ann/PI tubes (compare percentages in LR regions of histograms in (B) with percentages in lower-left plots in (A). (C) The good correlation of live cell regions between Ann/PI and intracellular staining tubes is reproducible. CLL cells were treated with 10 μM deguelin, 1μg/ml fludarabine or the combination of both and cultured 24h with Ltk- and 3T3-CD40LG cells. Graphs show the correlation between percentage of Ann-/IP- cells and percentage of cells in the gated live region in the same Ann/PI tubes (solid symbols). The majority of gated cells in the live region were Ann-/IP- (mean±SD: 94.5±6.1 in samples cultured with Ltk- and 96.8±3.4 with 3T3-CD40LG). Replicates in the 5 intracellular staining tubes for each sample are very similar, and also have a good correlation with the percentage of cells in the live cell region in the corresponding Ann/IP tubes (compare solid and open symbols for each treatment condition). (D) Stains of p-AKT, p-p65 and c-Myc in live and apoptot [file pone.0154159.s003.tif]
