# Supplementary material for: Synergistic Activity of Deguelin and Fludarabine in Cells from Chronic Lymphocytic Leukemia Patients and in the New Zealand Black Murine Model
Source: PLoS One. 2016 Apr 21;11(4):e0154159. doi: 10.1371/journal.pone.0154159 (PMC4839760; doi:10.1371/journal.pone.0154159)
Supplement: S1 Table — (DOCX) [file pone.0154159.s004.docx]

| **CLL ID** | **^1^ IgVH** | **Age** | **Sex** | **^2^CD38** | **^2^ZAP70** | **Lymphocytes** | **Rai** | **Binet** | **^3^Cytogenetic** | **^4^ED50** | **Previously** |
| --- | --- | --- | --- | --- | --- | --- | --- | --- | --- | --- | --- |
|  | **Status** |  |  |  |  | **cel/µl** |  |  | **alterations** | **(µM)** | **treated** |
|  |  |  |  |  |  |  |  |  |  |  |  |
| 1 | U | 42 | f | 4 | 74 | 44380 | II | B | trisomy 12; chr 14 | 15.03 | Yes |
| 2 | ^5^ND | 74 | m | 11 | 4 | 16950 | II | A | ND | 17.45 | No |
| 3 | ND | 80 | m | 1.5 | 10 | 25620 | 0 | A | 13q del | 7.13 | No |
| 3 | M | 78 | f | 81 | 83 | 16300 | 0 | A | ND | 31.29 | No |
| 4 | M | 73 | m | 5 | 9 | 4480 | 0 | A | 13q del | 6.92 | No |
| 5 | ND | 78 | f | 55 | 76 | 3850 | I | A | ND | 33.83 | No |
| 6 | M | 70 | m | 0.3 | 40 | 15450 | I | A | 13q del | 4.88 | No |
| 7 | U | 82 | m | 10 | 31 | 89980 | II | A | 11q del; 13q del | 48.84 | Yes |
| 8 | M | 75 | f | 1.2 | 14 | 11300 | 0 | A | 13q del | 6.03 | No |
| 9 | ND | 51 | m | 4 | 30 | 7020 | 0 | A | 13q del | 9.37 | No |
| 10 | U | 65 | m | 68 | 61 | 12090 | 0 | A | 13q del | 6.14 | No |
| 11 | M | 68 | m | 92 | 60 | 36760 | II | A | trisomy 12 | 3.50 | No |
| 12 | ND | 86 | f | 27 | 15 | 5090 | 0 | A | ND | 3.74 | No |
| 13 | ND | 59 | f | 34 | 50 | 7490 | 0 | A | Normal | 7.50 | No |
| 14 | M | 75 | f | 0.5 | 6 | 45970 | I | A | 13q del | 1.84 | Yes |
| 15 | ND | 52 | m | 11 | 23 | 8890 | 0 | A | 13q del | 5.14 | No |
| 16 | ND | 66 | f | ND | 9 | 8500 | 0 | A | 13q del | 10.62 | No |
| 17 | ND | 70 | m | 17 | 48 | 36010 | II | A | 13q del | 1.82 | Yes |
| 18 | M | 79 | m | 4 | 10 | 12770 | 0 | A | 13q del | 3.13 | No |
| 19 | ND | 62 | m | 4 | 7 | 11550 | 0 | A | 13q del | 1.34 | No |
| 20 | M | 86 | m | 1 | 26 | 18600 | 0 | A | ND | 4.34 | No |
| 21 | U | 73 | m | 12 | 57 | ND | I | A | ND | 9.25 | No |
| 22 | M | 87 | f | 0.2 | 6 | 22140 | I | A | ND | 7.11 | No |
| 23 | M | 95 | m | 20 | 19 | ND | ND | ND | ND | 0.18 | No |
| 24 | M | 70 | m | 15 | 19 | 5930 | 0 | A | 13q del | 7.73 | No |
| 25 | ND | 37 | m | 31 | 23 | 8580 | 0 | A | Normal | 4.82 | No |
| 26 | M | 66 | f | 4 | 14 | 14450 | 0 | A | 13q del | 3.82 | No |
| 27 | M | 60 | m | 7 | 43 | 17980 | 0 | A | Normal | 12.93 | No |
| 28 | M | ND | m | 0.7 | 23 | 6370 | 0 | A | ND | 7.04 | No |
| 29 | M | 86 | m | 1 | 26 | 18600 | 0 | A | ND | 4.34 | No |
| 30 | M | ND | f | 0.3 | 87 | 21800 | 0 | A | ND | 4.41 | No |
| 31 | M | 64 | f | 0.7 | 8 | 1690 | 0 | A | 13q del | 0.39 | No |
| 32 | M | 52 | m | 5 | 13 | 56920 | 0 | A | 13q del | 0.03 | Yes |
| 33 | ND | 74 | f | 5 | 15 | 8530 | 0 | A | 13q del | 2.07 | No |
| 34 | U | ND | m | 40 | 60 | ND | ND | ND | ND | 7.98 | No |
| 35 | M | 71 | f | 2 | 16 | 4890 | 0 | A | 13q del | 6.17 | No |
|  |  |  |  |  |  |  |  |  |  |  |  |
|  |  |  |  |  |  |  |  |  |  |  |  |

**Table S1. Patient characteristics.**

^1^IGHV sequence is considered mutated (M) when its homology to the germline IGHV sequence is < 98%. (U, unmutated).

^2^ Percentage of positive cells by flow cytometry.

^3^ Determined by interphase fluorescence *in situ* hybridization.

^4^ ED50 deguelin calculated by non-linear regression using Prism 5 software.

^5^ No determined
